# Supplementary figures and images for: Neurodegenerative Changes in the Brains of the 5xFAD Alzheimer’s Disease Model Mice Investigated by High-Field and High-Resolution Magnetic Resonance Imaging and Multi-Nuclei Magnetic Resonance Spectroscopy
Source: Int J Mol Sci. 2023 Mar 7;24(6):5073. doi: 10.3390/ijms24065073 (PMC10049146; doi:10.3390/ijms24065073)

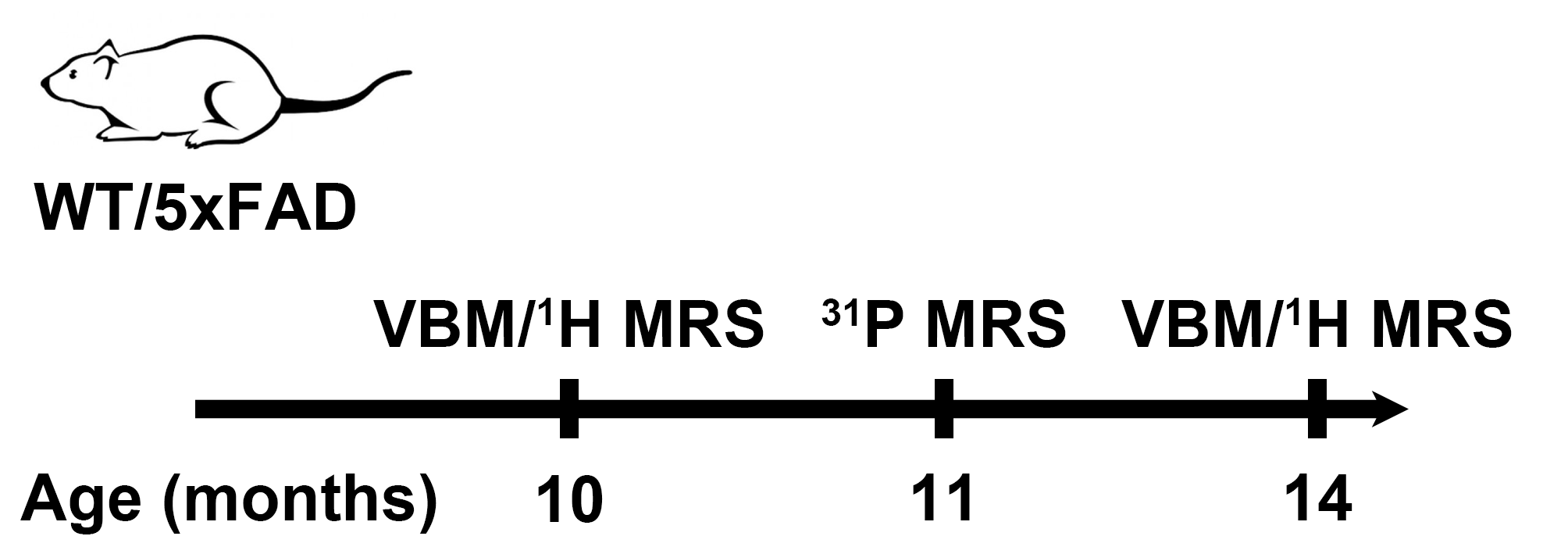

Supplement: Supplementary file 1 [file ijms-24-05073-s001.zip › ijms-2250533-supplementary.tif]
